# The Effectiveness of *¡Salud!, por la Vida*, an Educational Intervention to Increase Colorectal Cancer Screening in Puerto Rico

**DOI:** 10.3390/cancers17203391

**Published:** 2025-10-21

**Authors:** Josheili Llavona-Ortiz, Maria E. Fernández, Ileska M. Valencia-Torres, Francisco J. Muñoz-Torres, Marievelisse Soto-Salgado, Yara Sánchez-Cabrera, Vivian Colón-López

**Affiliations:** 1 Department of Public Health Sciences, Penn State University College of Medicine, Hershey, PA 17033, USA; jllavonaortiz@arizona.edu; 2 The University of Arizona Cancer Center, The University of Arizona, Tucson, AZ 85724, USA; 3 Center for Health Promotion and Prevention Research, The University of Texas Health Sciences Center at Houston, Houston, TX 77030, USA; maria.e.fernandez@uth.tmc.edu (M.E.F.); ileska.m.valenciatorres@uth.tmc.edu (I.M.V.-T.); 4 Division of Cancer Control and Population Sciences, The University of Puerto Rico-Comprehensive Cancer Center, San Juan, PR 00936, USA; francisco.munoz@upr.edu (F.J.M.-T.); marievelisse.soto1@upr.edu (M.S.-S.); yara.sanchez@upr.edu (Y.S.-C.)

**Keywords:** colorectal cancer, behavioral intervention, Federally Qualified Health Centers, Puerto Rico

## Abstract

**Simple Summary:**

Colorectal cancer is the leading cause of cancer-related death in Puerto Rico, yet screening rates remain low. To address this gap, researchers developed and tested a culturally tailored educational intervention, *¡Salud! Por la Vida*, aimed at increasing colorectal cancer screening among adults aged 50–75 who were not up to date with current guidelines. A randomized controlled trial was conducted in Federally Qualified Health Centers. Participants in the intervention group received one-on-one education from Community Health Workers (CHWs) along with follow-up reminders, while the control group received standard care. After four months, screening rates—including fecal occult blood tests and colonoscopies—were 48% higher among those in the intervention group. These findings suggest that CHW-delivered, culturally responsive strategies can effectively improve screening uptake in underserved populations. This study may inform future community-based cancer prevention initiatives and reduce late-stage diagnoses and mortality, contributing meaningfully to public health and health equity efforts.

**Abstract:**

**Background/Objectives**: Colorectal cancer (CRC) is the leading cancer-related death in Puerto Rico (PR). Yet CRC screening (CRCS) rates remain low. We developed *¡Salud!, por la Vida*, an educational intervention aiming to increase CRCS among age-eligible adults living in PR. **Methods**: We conducted a cluster randomized controlled trial among adults 50–75 years old at Federally Qualified Health Clinics in PR. Participants could not have a history of CRC nor be currently adherent to CRCS guidelines for a fecal occult blood test (FOBT) or fecal immunochemical test (FIT) (within last year) or colonoscopy (within last 5–10 years). Out of 445 randomized participants, 355 completed the study procedures (Control: 277; Intervention: 78) and were included in the main analysis. Participants in the intervention arm completed baseline and follow-up questionnaires alongside the educational intervention (at baseline) and two reminder calls (before follow-up) within a four-month period. Control arm participants only completed baseline and follow-up questionnaires within the same period. All participants were followed up to assess CRCS completion. **Results**: Post-trial screening rates were significantly higher in the intervention group: FOBT/FIT (55% vs. 39%, *p* = 0.02), colonoscopy (10% vs. 3%, *p* = 0.02), and any CRCS (60% vs. 41%, *p* < 0.01). Compared to controls, those in the intervention group showed a 48% higher probability of undergoing any CRCS (RR = 1.48, 95%CI: 1.17, 1.86), were 1.4 times more likely to complete a FOBT/FIT (RR = 1.40, 95%CI: 1.09, 1.80), and were over 3 times more likely to undergo a colonoscopy (RR = 3.16, 95%CI: 1.26, 7.91). **Conclusions**: The findings underscore the efficacy of the intervention in increasing CRCS uptake, potentially preventing late-stage detection and reducing CRC mortality in PR.

## 1. Introduction

Colorectal cancer (CRC) has remained among the ten most diagnosed cancers in Puerto Rico (PR), a United States (U.S.) territory with approximately 3.3 million residents, for over a decade [1,2]. The most recent report from the PR Central Cancer Registry ranked CRC as the second most common incident cancer (35 cases per 100,000 people) from 2016 to 2020 [2]. Disparities in advanced stages of CRC are particularly pronounced among socioeconomically disadvantaged populations on the island [3]. Late-stage CRC has also been associated with diagnostic delay (defined as more than 60 days between initial healthcare contact and diagnosis) and with receiving a CRC diagnosis during an emergency department visit [4].

Although survival rates decline for patients diagnosed at later stages, most CRC cases could be prevented through regular screening [5]. Despite strong evidence that CRC screening reduces overall mortality rates in both men and women, underutilization of screening methods such as fecal occult blood tests (FOBT), fecal immunochemical tests (FIT), colonoscopies, or flexible sigmoidoscopies, remains a concern [5,6,7].

Colorectal cancer screening (CRCS) rates in PR have consistently fallen below the national target goals of 80%, as set by Healthy People 2030 [8]. According to 2018 estimates from the Behavioral Risk Factor Surveillance System (BRFSS), the CRCS rate in PR was 58%, significantly lower than the national median of 70%, placing PR among the lowest-ranked regions in the United States [9]. Furthermore, the Health Resources and Services Administration (HRSA), which is the funding source for Federally Qualified Health Centers (FQHCs), reported that CRCS rates across FQHCs in PR fluctuated between 40.8% and 55.5% from 2019 to 2023, thus presenting even lower CRCS rates than those observed through the BRFSS [10].

Increasing CRCS was a goal of Healthy People 2020 and remains a priority in the updated Healthy People 2030 objectives [11,12]. To achieve this goal, efforts to identify and develop tailored interventions to increase CRCS rates have been ongoing. The *Guide to Community Preventive Services*, developed by the Community Preventive Services Task Force (CPSTF), recommends several evidence-based approaches to increase CRCS uptake. These include the use of small media, one-on-one client education, reminders, and provider assessment and feedback to increase CRCS [13].

A review found that interventions involving community health workers significantly increased CRC screening rates, with a median increase of 10.5%, regardless of race, ethnicity, income, or insurance status [14]. While evidence suggests that these approaches may increase CRCS, few studies have specifically targeted interventions for Hispanics populations [15], and none have focused primarily on Puerto Ricans, either in the U.S. mainland or in PR.

In this study, we conducted a group randomized controlled trial (RCT) in nine FQHCs in PR to evaluate the effectiveness of an educational intervention in increasing CRCS completion among Hispanic adults attending these centers. We hypothesized that patients who received the CRCS educational intervention would have higher CRCS completion rates compared to those in the control group. Considerations during the implementation of this study, regarding the impact of natural disasters (e.g., Hurricanes Irma and María, earthquakes) and the COVID-19 pandemic on this study, are also described below.

## 2. Materials and Methods

### 2.1. Intervention Design of ¡Salud!, por la Vida

The educational intervention *¡Salud!, por la Vida* (SPLV) was developed based on formative research using Intervention Mapping (IM) [16,17]. Briefly, IM serves as a planning framework to systematically define how to develop, implement, and evaluate a defined intervention [17]. In previous research by our team, a total of seven focus groups were involved in rural and urban FQHCs in PR to explore knowledge, attitudes, and beliefs regarding CRC and CRCS [18]. Participants identified several factors influencing their CRCS status, including lack of knowledge, low perceived risk, and barriers such as transportation issues, costs of screening tests, time constraints, and cultural beliefs. These insights guided the application of IM to develop a health behavior intervention targeting CRC and CRCS in Puerto Rico [19].

Following this formative assessment, the research team incorporated the fundamentals of entertainment education and behavioral journalism to develop a tailored interactive multimedia intervention (TIMI) that addresses common barriers and concerns about CRC and CRCS among Puerto Rican patients [19]. The TIMI presents the story of a Puerto Rican couple planning their wedding anniversary but decides to postpone their plans after a close friend is diagnosed with CRC. Their friend’s diagnosis prompts them to visit their doctor and inquire about screening. As the story unfolds, participants navigate through a series of short video clips addressing concerns, barriers, and perceived risks associated with CRC. These video clips are tailored based on the participant’s sex and family history of CRC.

This study employed a cluster RCT design with one control arm and one intervention arm to evaluate the efficacy of SPLV in improving CRCS uptake in age-eligible patients across FQHCs in Puerto Rico [20]. Therefore, the arm assignment occurred at the clinic level. Once a clinic was randomized to either the intervention or control arm, eligible patients within the clinic would automatically be assigned to the clinic arm. The study was approved by the University of Puerto Rico-Medical Sciences Campus Institutional Review Board IRB# 2290034115 and is registered in Clinicaltrials.gov (NCT05502666).

#### *¡Salud!, por la Vida* (SPLV) Components

The SPLV intervention consisted of a TIMI, healthcare provider and patient prompts, and printed educational materials, including infographics and a newsletter (Table 1). Participants assigned to the intervention arm complete the TIMI after answering a baseline questionnaire. Upon completing the TIMI, participants received a printed provider prompt indicating their intent regarding CRCS: (1) “I will definitely get screened to prevent CRC”, (2) “I am thinking about getting screened for CRC”, or (3) “I am not interested in getting screened for CRC.”).

When handing over the printed prompt, the CHW encouraged participants to use this document as an icebreaker when discussing their CRCS intentions with their healthcare provider. Participants also received a tote bag containing informational flyers about on the FOBT/FIT, colonoscopy, a step-by-step guide on preparing for the CRCS tests, and a general summary of CRC and CRCS guidelines. All SPLV materials were developed in Spanish.

### 2.2. FWHC Clinic Selection and Sampling

Recruitment efforts were designed to achieve a balanced sample across intervention and control groups, following a cluster-randomized design. Eligibility criteria were established based on prior studies, anticipating a 20% screening completion rate in the control group associated with the minimal intervention cues provided in the comparison condition (a prompt to receive CRCS), and a 15% increase in the intervention group. Inclusion criteria were: (1) age between 50 and 75 years; (2) currently receiving medical services at a participating FQHC; (3) no personal history of colorectal cancer; (4) no FOBT or FIT completed in the past year; and (5) no colonoscopy completed in the past 10 years (or 5 years if family history was reported).

**Figure 1:**
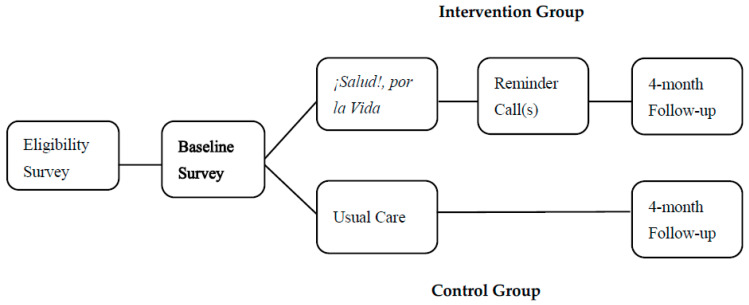
Study design.

Given variations in patient flow across clinics, the study design incorporated flexibility to allow higher enrollment in clinics with greater recruitment capacity while maintaining overall group balance (Table 2). Based on our power analysis results, the sample size was adequate to be sufficient to detect meaningful differences in CRC screening rates between the intervention and control groups (See Figure 1).

### 2.3. Baseline and Follow-Up Instruments

The baseline questionnaire explored demographic and cancer screening practices. In addition to demographic variables, the questionnaire included the following topics: CRC knowledge, family history of CRC, FOBT/FIT history, colonoscopy history, perceived risk, self-efficacy for completing CRCS, perceived advantages and disadvantages of CRCS, subjective norms, social norms, healthcare utilization, and overall perceived health [21,22,23,24].

For the FOBT/FIT and colonoscopy history questions, participants who had previously completed at least one CRCS test but were currently not currently up to date were asked to indicate reasons for not maintaining screening adherence. Participants who had never undergone CRCS tests were asked to select all the reasons that prevented them from doing so and to identify the primary reason for non-compliance.

The follow-up questionnaire assessed the same topics as the baseline questionnaire, excluding demographic variables, family history of CRC, and screening questions specific to women (e.g., cervical cancer and breast cancer screening behaviors). In addition, participants were asked whether they had completed an FOBT/FIT or colonoscopy since the baseline assessment. Depending on their responses, they were either asked to detail the steps taken toward screening or to indicate reasons for nor-completion.

Participants in the intervention arm also responded to specific questions regarding their experience with SPLV. These questions assessed whether they identified with the actors in the video, found the educational materials useful, perceived the information provided as easy to understand, and whether the intervention influenced their decision to undergo a CRCS test. Participants also rated their overall satisfaction with SPLV.

### 2.4. Study Implementation

A collaborative agreement was established with the *Asociación de Servicios de Salud Primaria de PR* (Primary Health Services Association of Puerto Rico), a non-profit organization supporting FQHCs across PR. Memorandums of understanding (MOUs) were signed with all participating centers to: (1) establish multiple weekly site visits for in-person recruitment, (2) identify private areas to guarantee participant confidentiality, and (3) define HIPAA-compliant data-sharing policies for obtaining CRCS completion data from consenting participants.

The RCT was conducted in nine FQHCs, which were randomly assigned to either the intervention arm (*n* = 4 centers) or the control arm (*n* = 5 centers). Stratified randomization procedures were used to ensure that clinics in both arms had comparable patient volumes (e.g., similar estimated number of patients seen per year) and availability of colorectal screening services (e.g., laboratory embedded vs. non-laboratory embedded clinics to process FOBT/FIT findings). Clinics were first grouped (stratified) based on these key characteristics, and then randomly assigned to either the intervention or control group within each stratum. This approach helped minimize potential confounding factors related to clinic-level differences. Between 11 and 78 participants were randomly recruited from each participating center and included in the study. Figure 1 illustrates the study design. After randomization, a baseline survey was administered in both intervention and control groups, with follow-up assessments conducted four months after baseline [21,22,23,24,25].

#### Participant Recruitment and Data Collection

Recruitment took place from 1 August 2017 to 6 March 2020. Participants recruited were patients aged 50–75 receiving medical services at one of the nine participating FQHCs at the time of enrollment. Eligibility criteria required that participants had no personal history of CRC and had not completed an FOBT or FIT in the last year or a colonoscopy in the past 10 years (or 5 years if family history was reported) (Figure 1).

Trained CHWs administered a screening questionnaire to all potential participants. If eligible, the CHW provided a brief overview of the study to assess the patient’s interest in participating. Eligible individuals were asked to sign a consent form before completing the baseline questionnaire.

Participants in the intervention arm were provided with a touchscreen tablet to complete the TIMI after completing the baseline questionnaire. Upon completing the TIMI, they received a provider prompt to facilitate discussions with their primary care physician about CRCS. All participants received a $20 gift card after completing the baseline questionnaire.

Participants in the control arm received a follow-up phone call four months after recruitment to complete a follow-up questionnaire. Participants in the intervention arm received two additional phone calls—one week and one month after recruitment—before their follow-up questionnaire to assess their CRCS status. Participants in both arms who completed the four-month follow-up questionnaire were provided with a $20 gift card.

To minimize loss to follow-up, multiple strategies were implemented. Follow-up phone calls were made at different times of the day and to secondary contacts (if provided). Additionally, non-respondents received mailed letters prompting them to contact the study team. Lastly, clinical records of participants were requested from participating centers following HIPAA-compliant procedures to obtain data on CRCS completion (primary outcome). Secondary outcomes included changes in knowledge, attitudes, self-efficacy, and intentions related to CRCS [21,22,23,24].

Among 5112 initially screened individuals, 445 (9%) met the study’s inclusion criteria. The primary reasons for ineligibility were being up to date with CRCS tests (*n* = 3700; 72.3%), refusal to complete the eligibility assessment form (*n* = 482; 9.4%), or other reasons such as lack of time (*n* = 247; 4.8%). A total of 113 participants were assigned to the SPLV intervention group, and 332 were assigned to the control group. Of these, 78 (69%) in the intervention group and 277 (83%) in the control group completed assessments, resulting in a final analytic sample of 355 participants. Baseline characteristics of the 445 initially included participants, before excluding those with incomplete study assessments, are presented in Supplementary Table S1.

## 3. Results

The statistical power for the primary outcome, “Either Screening,” is estimated at 0.86, exceeding the conventional 0.80 threshold for sufficient power. This aligns with our original study design, which aimed to detect a 15% difference in screening completion with 80% power at α = 0.05. Since our achieved power surpasses this target, the sample size effectively supports the validity of our findings, ensuring that the study was adequately powered to detect the expected intervention effects.

Table 3 presents characteristics of the study participants overall, as well as within the intervention and control groups. No statistically significant differences were observed between the intervention and control group arms in terms of age, education, marital status, or income (*p* > 0.05). On average, participants had a mean age of 59.3 years, with approximately 70% having completed 12 years of education or less. Over half (52%) of the participants reported an annual income of $5000 or less. However, differences between the groups were observed when comparing the intervention (SPLV) to the control group. The SPLV intervention group showed a higher prevalence of individuals without insurance coverage (17% vs. 3%, *p* < 0.001).

Among all participants who completed the baseline and follow-up assessments, 152 (43%) reported completing the FOBT, 17 (5%) underwent a colonoscopy, and 9 (3%) reported having completed both screenings. Comparing the SPLV intervention group to the control group revealed higher rates in the former across all three outcomes: FOBT (55% vs. 39%, *p* = 0.02), colonoscopy (10% vs. 3%, *p* = 0.02), and either screening (60% vs. 41%, *p* < 0.01).

Table 4 shows a significant difference in first-degree family history of CRC between study groups (*p* = 0.02; 20.8% in the intervention group vs. 10.3% in the control group). Previous screening tests, including the fecal occult blood test (FOBT) and colonoscopy, showed no significant differences between the groups.

Participants in both the intervention and control groups generally expressed positive attitudes towards CRC screening, with a majority in each group indicating a definite intention to undergo testing (48.7% in the intervention and 38.8% in the controls, *p* = 0.26). However, a significant difference was observed between the SPLV intervention and control groups regarding the reported probability of undergoing a CRC test in the next three months (*p* = 0.04).

Table 5 presents the reported perceived risk of developing colorectal cancer (CRC) among the SPLV and control groups. There were no statistically significant differences in the perceived likelihood of developing CRC at some point in life or within the next five years.

Table 6 provides an overview of psychosocial variables at baseline, comparing the intervention and control groups. The table includes measures of knowledge, perceived risk, self-efficacy, subjective norms, and social norms, presenting both numerical counts and percentages for knowledge and medians with minimum and maximum values for the other variables.

Statistically significant differences were observed between the groups in perceived risk of developing CRC and subjective norms (*p* = 0.04). However, knowledge about CRCS, self-efficacy, and social norms showed no statistically significant differences between the intervention and control groups.

Compared to the control group (Table 7), the SPLV intervention group had a 48% higher probability of undergoing either CRC screening (RR = 1.48, 95% CI: 1.17, 1.86); a 40% higher probability of completing FOBT/FIT (RR = 1.40, 95% CI: 1.09, 1.80); and more than three times the probability of undergoing a colonoscopy (RR = 3.16, 95% CI: 1.26, 7.91).

## 4. Discussion

This study aimed to evaluate the effectiveness of *¡Salud!, por la Vida*, a culturally tailored educational intervention designed to promote CRCS among Spanish-speaking Hispanics attending FQHCs in PR. SPLV significantly increased the likelihood of completing CRCS, including stool-based tests (FOBT or FIT) and colonoscopies. Participants in the intervention group were nearly 50% more likely to complete at least one CRCS test than those in the control group. Among previously non-adherent individuals, those in the intervention group were over three times more likely to undergo a colonoscopy than controls. These findings align with other studies showing that educational interventions, particularly those delivered by CHWs, can enhance CRCS uptake in underserved populations [15,26,27]. Although a significant difference in the first-degree family history of CRC was observed between the study groups, models adjusted for family history of CRC yielded similar results compared to the other models [28].

At baseline, our analysis of psychosocial variables revealed significant differences between the intervention and control groups in perceived risk and subjective norms related to CRCS. While median values for these variables were similar across both groups, the intervention group reported slightly higher perceived risk and stronger subjective norms in favor of CRCS, which may have influenced their screening behavior. However, other psychosocial factors, such as knowledge about CRC screening, self-efficacy, and social norms, did not differ significantly between groups. Additionally, the magnitude of the associations in the screening models remained unchanged after including these variables in the models. This suggests that the intervention’s impact on screening behaviors was primarily driven by its content and delivery rather than by baseline psychosocial differences.

The success of SPLV in promoting CRCS can be attributed to several key factors. First, the intervention was developed using a systematic framework grounded in evidence-based strategies, and was culturally adapted to address the unique needs and barriers faced by the Puerto Rican population [18,19]. Culturally adapted interventions help address cultural, behavioral, and environmental factors that influence screening by tackling barriers specific to the target population [29,30]. In addition to cultural adaptation, the SPLV intervention benefited from being delivered by trained CHWs. Research shows that one-on-one education provided by Lay Health Workers (LHWs) and CHWs is effective in promoting cancer prevention and increasing screening behaviors and adherence [13,31,32,33]. A recent systematic review of 76 studies found that LHW-delivered interventions could increase colorectal cancer screening rates by a median of 10.5 percentage points (interquartile interval: 4.5 to 17.5) [34].

Another contributing factor may have been the use of provider prompts, which have been identified as one of the top evidence-based interventions selected by healthcare systems participating in the Colorectal Cancer Control Program in the U.S. to increase CRCS uptake [35]. Each SPLV intervention participant received a personalized, printed provider prompt and was instructed to share it with their provider at their next appointment. Since participants were recruited at clinics while waiting to receive healthcare services, these prompts—combined with the educational intervention—may have served as an icebreaker, facilitating conversations about CRCS.

By documenting and demonstrating the effectiveness of this culturally relevant intervention for Puerto Ricans, our study contributes to ongoing research efforts aimed at understanding and enhancing tailored interventions for Hispanic communities. To our knowledge, this is the first study to develop and evaluate an evidence-based intervention specifically designed to increase CRCS uptake among Puerto Ricans. Findings highlight the importance and impact of culturally tailored interventions in increasing CRCS, as they engage the target population through culturally relevant components.

Given the high CRC mortality rates and low screening rates in PR, implementing culturally adapted interventions could be a key strategy for increasing screening and follow-up care. The SPLV intervention significantly increased CRC screening rates, providing strong evidence for disseminating this educational effort across all FQHCs and other clinical settings in PR. Future efforts should focus on testing and culturally adapting the intervention for U.S.-based Hispanics and other groups at a high risk of CRC.

### Limitations and Related Considerations

This study was conducted from August 2017 through July 2020, during which study implementation was affected by three natural disasters and a pandemic. On September 6 and 20, 2017, Hurricanes Irma and María impacted PR as a Category 5 and Category 4 hurricane, respectively. These hurricanes left the island without communication, utility services, or adequate access to healthcare services [36,37,38]. In response, the President of the United States issued two Major Disaster Declarations in September 2017 [39,40], leading to a temporary pause in all research activities. Recruitment activities resumed in control clinics in January 2018; however, clinics and patients were still recovering, which led to ongoing challenges in communication, transportation, clinic visits, and study participation. Research activities at intervention clinics were reinstated in May 2019.

In December 2019, a third Major Disaster Declaration was issued due to an earthquake swarm that lasted eight months, causing more than 4000 aftershocks, primarily affecting the southern and southwestern regions of the island [41,42,43,44]. These events disrupted both clinic and research activities, including CRCS services and participants follow-ups, as clinics reduced hours or temporarily halted services. This event also affected follow-up efforts by phone, as some participants were hesitant to continue the study due to concerns about spam calls.

Three months later, the U.S. government issue a fourth Major Disaster Declaration in response to the COVID-19 pandemic [45]. This led to widespread shutdowns, limited in-person activities, healthcare understaffing, increased healthcare demands, economic turmoil, and missed screening opportunities, among other situations. Other studies conducted in Puerto Rico have documented how these events disrupt cancer screening services [18,46]. Overall, these disruptions affected the research team’s ability to complete participant follow-ups due to changes in phone numbers and address, as well as participants relocating outside the country.

Technological delays in developing the intervention further complicated recruitment. The intervention took approximately two years to finalize and was not fully launched until May 2019, creating a gap between the design phase and implementation. These delays also made it challenging to recruit participants for both the intervention and control arms simultaneously. Consequently, it is unclear whether socioeconomic and historical factors for the intervention group changed significantly over time compared to when control group participants were recruited.

Another limitation relates to the accuracy of self-reported screening status. While participants were asked about their adherence to CRC screening guidelines, self-reports may have been inaccurate due to confusion about specific tests or misreporting of screening completion. To mitigate this, clinical records were reviewed up to a year following baseline enrollment, allowing for a more accurate comparison between self-reported and actual screening data. Clinical record review also helped address limitations in data collection for the primary outcome (CRCS uptake) in cases where follow-up was not possible. One of the challenges reported by participants was uncertainty about whether they would have the economic means to purchase additional minutes for their prepaid phones by the time follow-up was due. Despite these limitations, this study shows a significant impact of the *!Salud! por la Vida* intervention on CRCS rates. The findings of this study underscore the importance of interventions tailored to the realities, culture, and needs of communities disproportionately affected by cancer, in this case, CRC.

## 5. Conclusions

This study provides evidence that culturally tailored educational interventions can significantly improve CRC screening rates. By integrating CHW one-on-one education, provider prompts, and culturally relevant materials, the intervention led to increased CRC screening among participants. These findings reinforce the importance of culturally adapted interventions in reducing health disparities and improving cancer prevention efforts in underserved communities.

Despite challenges posed by natural disasters, a global pandemic, and technological delays, SPLV remained effective, demonstrating resilience in real-world conditions. The study’s robust methodology, including clinical record verification, strengthens the validity of our results. However, future research should explore additional strategies to mitigate self-reporting biases and recruitment challenges.

Given SPLV’s success, expanding this intervention to other subgroups and high-risk populations could further enhance CRC screening uptake and reduce disparities in cancer prevention. Recent findings also indicate that Hispanic/Latino community members in the U.S. are interested in community health worker-led interventions aiming to address existing barriers to CRCS [47]. Future efforts should also explore bundling CRCS with other preventive services to maximize engagement and long-term adherence [48,49,50]. By continuing to implement and refine culturally tailored interventions, we can make meaningful strides in cancer prevention and early detection by addressing screening uptake, follow-up procedures, and key health cancer-related knowledge for our communities.

## Figures and Tables

**Table 1:** An overview of the educational materials designed to inform and guide individuals through the colorectal cancer screening process.

Educational Material	What Was Provided/Explained
Colonoscopy Process	Details on the colonoscopy procedure, including the steps involved, the action plan for undergoing the procedure, and the challenges that might arise during the process.
Blood Test with Labs	Blood test process that requires lab involvement, covering how the test works and the action plan for completing the test for occult blood in stool.
Blood Test without Labs	Blood test that does not require lab work and outlines the action plan for completing the stool occult blood test.
Infographic tailored for Women	Featuring a testimony from a woman discussing colorectal cancer and the importance of screening tests.
Infographic tailored for Men	Highlighting colorectal cancer and screening, with relevant information and a testimony from a man.
Steps for Completing the FOBT	A step-by-step diagram showing how to collect and return the stool sample for the fecal occult blood test (FOBT).

**Table 2:** Implementation strategies for the SPLV intervention, and how recruitment and participant engagement was managed to ensure successful enrollment and participation.

Strategy	Description
Recruitment via Banner	A banner displaying key study details (title, short description, eligibility criteria, and contact information) was posted in the waiting area of FQHCs. This helped inform potential participants about the study.
In-Person Recruitment	Trained staff visited FQHCs multiple times to screen patients for eligibility in person. If patients were eligible and interested, staff would provide further study details and the informed consent form in English or Spanish, depending on the individual’s preference.
Recruitment via Phone	Interested individuals who saw the banner or heard about the study were able to contact the study coordinator by phone to learn more and check eligibility. If eligible, an appointment was scheduled for the recruitment process.
Pre-Visit Screening List	The study team would receive a list of scheduled patients who met the basic eligibility criteria (age and sex) from each FQHC. This list helped plan recruitment visits by scheduling specific days and times for each clinic.
Flexible Appointment Scheduling	Participants were offered a range of appointment times, including mornings, afternoons, and evenings on weekdays. The study coordinator would ensure participants could select the most convenient time.
Informed Consent Process	Participants were notified in advance that they would need to sign a consent form before participating. The consent process was conducted at FQHCs, and each participant was given time to review (or have read to them) the form before signing.
Follow-Up Reminder	At least one phone call was made to each participant as a reminder prior to the recruitment visit, ensuring they were prepared for the study process.

**Table 3:** A description of the demographic characteristics of the *¡Salud!, por la Vida* (SPLV) study overall and by study group.

	Total n (%)	Intervention n (%)	Control n (%)	*p*-Value
Sample Size	355 (100)	78 (22.0)	277 (78.0)	
Sex				
Female	247 (69.6)	58 (74.4)	189 (68.2)	0.37
Male	108 (30.4)	20 (25.6)	88 (31.8)	
Age (mean ± SD)	59.3 ± 6.1	59.3 ± 6.1	59.2 ± 6.1	0.99
Education				
≤12th grade	247 (69.6)	50 (64.1)	197 (71.1)	
Post-secondary education	108 (30.4)	28 (35.9)	80 (28.9)	0.29
Marital status				
Never married	61 (17.2)	12 (15.4)	49 (17.7)	0.74
Married or living together	164 (46.2)	39 (50.0)	125 (45.1)	
Divorced, separated, or widowed	130 (36.6)	27 (34.6)	103 (37.2)	
Income per year				
≤$5000	183 (52.3)	37 (48.7)	146 (53.3)	0.63
$5000–$14,999	118 (33.7)	26 (34.2)	92 (33.6)	
≥$15,000	49 (14.0)	13 (17.1)	36 (13.1)	
Health insurance coverage				
Uninsured	21 (6.1)	13 (17.1)	8 (3.0)	<0.001
Insured	326 (93.9)	63 (82.9)	263 (97.0)	

**Table 4:** CRC family history, screening status, and attitudes by study groups.

	Intervention n (%)	Control n (%)	*p*-Value
First-degree family history of CRC			
Yes	16 (20.8)	28 (10.3)	0.02
No	61 (79.2)	244 (89.7)	
Prior FOBT			
Yes	49 (62.8)	201 (72.6)	0.13
No	29 (37.2)	76 (27.4)	
Prior Colonoscopy			
Yes	10 (12.8)	30 (10.8)	0.77
No	68 (87.2)	247 (89.2)	
Current plans to get CRCS			
Have not thought about it	5 (6.4)	30 (10.9)	0.26
Thinking about it	11 (14.1)	35 (12.7)	
Should get screened, but not ready	8 (10.3)	51 (18.5)	
I will probably get screened	16 (20.5)	53 (19.2)	
I will definitely get screened	38 (48.7)	107 (38.8)	
CRC test in the next 3 months			
Not probable	7 (9.2)	44 (16)	0.04
Somewhat probable	21 (27.6)	58 (21.1)	
Probable	20 (26.3)	105 (38.2)	
Highly Probable	28 (36.8)	68 (24.7)	

**Table 5:** Perceived risk of CRC by Study Group.

	SPLV n (%)	Control n (%)	*p*-Value
High lifetime CRC likelihood			
Completely Agree	27 (35.5)	107 (41.5)	0.08
Agree	15 (19.7)	67 (26)	
Disagree	25 (32.9)	49 (19)	
Completely Disagree	9 (11.8)	35 (13.6)	
Higher CRC likelihood within 5 years compared to peers			
Completely Agree	34 (43.6)	104 (37.5)	0.85
Agree	24 (30.8)	85 (30.7)	
Disagree	13 (16.7)	40 (14.4)	
Completely Disagree	7 (9)	31 (11.2)	
Higher lifetime CRC likelihood compared to peers			
Completely Agree	37 (47.4)	136 (49.1)	0.83
Agree	14 (17.9)	53 (19.1)	
Disagree	17 (21.8)	51 (18.4)	
Completely Disagree	10 (12.8)	27 (9.7)	

**Table 6:** Psychosocial Variables at Baseline by Study Group.

	SPLV *n* = 78	Control *n* = 277	*p*-Value
Knowledge *	31 (39.7)	111 (40.1)	1
Perceived Risk	3 (2, 4)	3 (1, 3)	0.04
Self-efficacy	27 (23, 30)	25 (22, 29)	0.13
Subjective norms	18 (16, 21)	17 (14, 19)	0.04
Social norms	6 (6, 7)	6 (5, 7)	0.43

* Knowledge yes/no, n (%), and Chi-Square *p*-value was used for analysis; for other variables in Table 6, median (Q1, Q3) and Mann–Whitney *p*-value were used.

**Table 7:** Risk Ratios and 95% Confidence Intervals for CRCS by SPLV Intervention vs. Control.

	SPLV	Control		
	CRCS/n (%)	CRCS/n (%)	RR_a_ (95% CI)	RR_b_ (95% CI)
Either Screening	47/78	113/277	1.48	1.44
	60.3	40.8	(1.17, 1.86)	(1.14, 1.82)
FOBT/FIT	43/78	109/277	1.40	1.37
	55.1	39.4	(1.09, 1.80)	(1.06, 1.76)
Colonoscopy	8/78	9/277	3.16	3.08
	10.3	3.2	(1.26, 7.91)	(1.22, 7.81)

a. Unadjusted. b. Adjusted for family history of CRC.

## Data Availability

Data are contained within the article/Supplementary Materials.

## Supplementary Materials

The following supporting information can be downloaded at https://www.mdpi.com/article/10.3390/cancers17203391/s1: Supplementary Table S1: Descriptives of ¡Salud!, por la Vida overall and by study group, based on the 445 participants who answered the questionnaire. Used to compare this sample to the 355 with complete data included in the paper.

## Abbreviations

The following abbreviations are used in this manuscript:

BRFSS	Behavioral Risk Factor Surveillance System
CRC	Colorectal cancer
CRCS	Colorectal cancer screening
FIT	Fecal immunochemical tests
FOBT:	Fecal occult blood tests
FQHC	Federally Qualified Health Centers
HRSA	Health Resources and Services Administration
IM	Intervention Mapping
RCT	Randomized controlled trial
SPLV	*¡Salud!, por la Vida*
TIMI	Tailored interactive multimedia intervention
