# Supplementary material for: The Effectiveness of ¡Salud!, por la Vida, an Educational Intervention to Increase Colorectal Cancer Screening in Puerto Rico
Source: Cancers (Basel). 2025 Oct 21;17(20):3391. doi: 10.3390/cancers17203391 (PMC12563970; doi:10.3390/cancers17203391)
Supplement: Supplementary file 1 [file cancers-17-03391-s001.zip › cancers-3808676-supplementary.pdf]

**Supplementary Material**

**Table S1.** Descriptives of *¡Salud!, por la Vida* overall and by study group, based on the 445 participants who answered the questionnaire. Used to compare this sample to the 355 with complete data included in the paper.

| | **Total N (%)** | **Intervention N (%)** | **Control N (%)** | **p-value** |
|---|---|---|---|---|
| **Sample Size** | 445(100) | 113 (23.4) | 332 (74.6) | |
| **Age (mean ± SD)** | 59.6 ± 6.3 | 59.4 ± 6.1 | 59.7 ± 6.3 | 0.99 |
| **Education** | | | | |
| ≤ $12^{th}$ grade | 317 (71.2) | 71 (62.8) | 246 (74.1) | |
| Post-secondary education | 128 (28.8) | 42 (37.2) | 86 (25.9) | 0.03 |
| **Marital status** | | | | |
| Never married | 75 (16.9) | 15 (13.3) | 60 (18.1) | 0.50 |
| Married or living together | 194 (43.6) | 51 (45.1) | 143 (43.1) | |
| Divorced, separated, or widowed | 176 (39.6) | 47 (41.6) | 129 (38.9) | |
| **Income per year** | | | | |
| ≤ $5,000 | 238 (54.6) | 52 (48.1) | 186 (56.7) | 0.28 |
| $5,000-$14,999 | 142 (32.6) | 39 (36.1) | 103 (31.4) | |
| ≥ $15,000 | 56 (12.8) | 17 (15.7) | 39 (11.9) | |
| **Health insurance coverage** | | | | |
| Uninsured | 23 (5.3) | 15 (13.9) | 8 (2.5) | < 0.001 |
| Insured | 409 (94.7) | 93 (86.1) | 316 (97.5) | |
| **First-degree family history of CRC** | | | | |
| Yes | 57 (13.1) | 22 (19.6) | 35 (10.8) | 0.03 |
| No | 379 (86.9) | 90 (80.4) | 289 (89.2) | |
